# Isolation, identification and antimicrobial susceptibility of the bacteria isolated from *Hyalomma dromedarii* infesting camels in Al-Jouf province, Saudi Arabia

**DOI:** 10.3389/fvets.2023.1227908

**Published:** 2023-12-14

**Authors:** Alanoud T. Aljasham, Eman M. Damra, Nora S. Alkahtani, Abdulaziz Alouffi, Waleed S. Al Salem, Aljoharah O. Alshabanah, Moureq Alotaibi, Tetsuya Tanaka, Abid Ali, Mashal M. Almutairi

**Affiliations:** ^1^Department of Pharmacology and Toxicology, College of Pharmacy, King Saud University, Riyadh, Saudi Arabia; ^2^Department of Agriculture, Ministry of Environment, Water, and Agriculture, Riyadh, Saudi Arabia; ^3^King Abdulaziz City for Science and Technology, Riyadh, Saudi Arabia; ^4^Laboratory of Infectious Diseases, Joint Faculty of Veterinary Medicine, Kagoshima University, Kagoshima, Japan; ^5^Department of Zoology, Abdul Wali Khan University Mardan, Mardan, Pakistan

**Keywords:** ticks, *Hyalomma dromedarii*, tick-borne bacteria, antimicrobial resistance, antimicrobial agents

## Abstract

Ticks are important ectoparasites that transmit various pathogens causing morbidity and mortality in humans and animals. Saudi Arabia faces several challenges that can contribute to the emergence and spread of antimicrobial resistance (AMR) bacteria. These challenges require collaborative efforts to successfully achieve significant control of AMR in the country. The present study aims to isolate bacteria from camels' tick *Hyalomma dromedarii* in Al-Jouf province to identify and determine these isolates' antimicrobial susceptibilities. Forty-nine ticks were collected from dromedary camels and morphologically classified as *H. dromedarii*. Ticks were then homogenized and plated individually, which resulted in the isolation of 55 bacteria. The results showed that the bacterial isolates belong to 20 different species. About 71% (*n* = 39) of the total isolates were identified as Gram-positive bacteria comprised of 11 different species, while 29% (*n* = 16) of the total isolates were Gram-negative bacteria comprised of 9 different species. The most prevalent isolate within the total samples was *Staphylococcus lentus* (22.45%, 11/49), followed by *Staphylococcus pseudintermedius* (18.37%, 9/49) and *Sphingomonas paucimobilis* (16.33% 8/49). The antimicrobial susceptibility profile of Gram-positive bacteria showed that 100% (*n* = 31) were resistant to benzylpenicillin; 90.3% (*n* = 28) were resistant to oxacillin; 58.1% (*n* = 18) were resistant to clindamycin; 48.4% (*n* = 15) were resistant to vancomycin. In addition, 32.3% (*n* = 10) were resistant to trimethoprim/sulfamethoxazole and rifampicin; 25.8% (*n* = 8) were resistant to erythromycin; 16.1% (*n* = 5) were resistant to teicoplanin; 6.5% (*n* = 2) were resistant to tetracycline. All Gram-positive bacteria were 100% susceptible to linezolid, gentamicin, tobramycin, levofloxacin, moxifloxacin, tigecycline, and nitrofurantoin. In antimicrobial susceptibility tests for the Gram-negative bacteria, 57.14% (*n* = 8) of the identified bacteria were resistant to ampicillin, whereas 50% (*n* = 7) were resistant to cefoxitin and ceftazidime. About 28.57% (*n* = 4) of the Gram-negative bacteria were resistant to ceftriaxone, trimethoprim/sulfamethoxazole. In addition, 21.43% (*n* = 3) were resistant to amoxicillin/clavulanic acid and cephalothin; 14.29% (*n* = 2) were resistant to cefepime and nitrofurantoin; 7.14% (*n* = 1) were resistant to piperacillin/tazobactam and tigecycline. However, all Gram-negative bacteria were susceptible to other examined antimicrobials. This is the first study that investigates the role of the hard tick as a potential reservoir for AMR pathogens within our region.

## 1 Introduction

Ticks are recognized as one of the main arthropods' vectors of disease agents to both humans and animals (1–3). Ticks can transmit a wide spectrum of pathogenic and non-pathogenic microorganisms such as bacteria, protozoa and viruses (4–6). Different bacteria have been detected in hard ticks at different developmental stages (7–10). Some microorganisms are life-threatening to animals (11), whereas others have risks to human health (12). The major losses caused by ticks are due to their ability to transmit diseases to livestock, which are of great economic importance worldwide. In addition, blood sucking by ticks causes anemia and reduction in weight among livestock, while their bites also reduce the quality of hides (13). These factors result in a substantial reduction in milk and meat production, increasing the morbidity and mortality among livestock.

Antimicrobial resistance (AMR) bacteria are microorganisms, mainly bacteria, that show resistance to one or more classes of antimicrobial agents (14). Multi-drugs resistance (MDR) are microorganism resistance to at least one antimicrobial in 3 or more different classes (15). The emergence and spread of AMR pathogens, which have acquired novel resistance mechanisms, is currently one of the most important threats to public and animal health. AMR is associated with a remarkable burden of increased morbidity, mortality, healthcare costs, and antibiotic use (15–17). AMR bacterial infections kill approximately 700,000 individuals globally each year, which is expected to rise to 10 million by 2050 (18). The AMR bacteria have been found in humans, animals, foods, plants, and the environment (water, soil, and air). They can spread from person to person or between humans and animals (19).

The present study aims to isolate and identify bacterial species from the camel's tick *H. dromedarii* in Al-Jouf province in Saudi Arabia. In addition, we aim to determine if the isolated bacterial species are resistant to clinical useful antimicrobial agents. To the best of our knowledge, this is the first report that investigates the hard tick's role as a potential reservoir for AMR bacteria in our region.

## 2 Materials and methods

### 2.1 Selection of the study area

**Figure 1:**
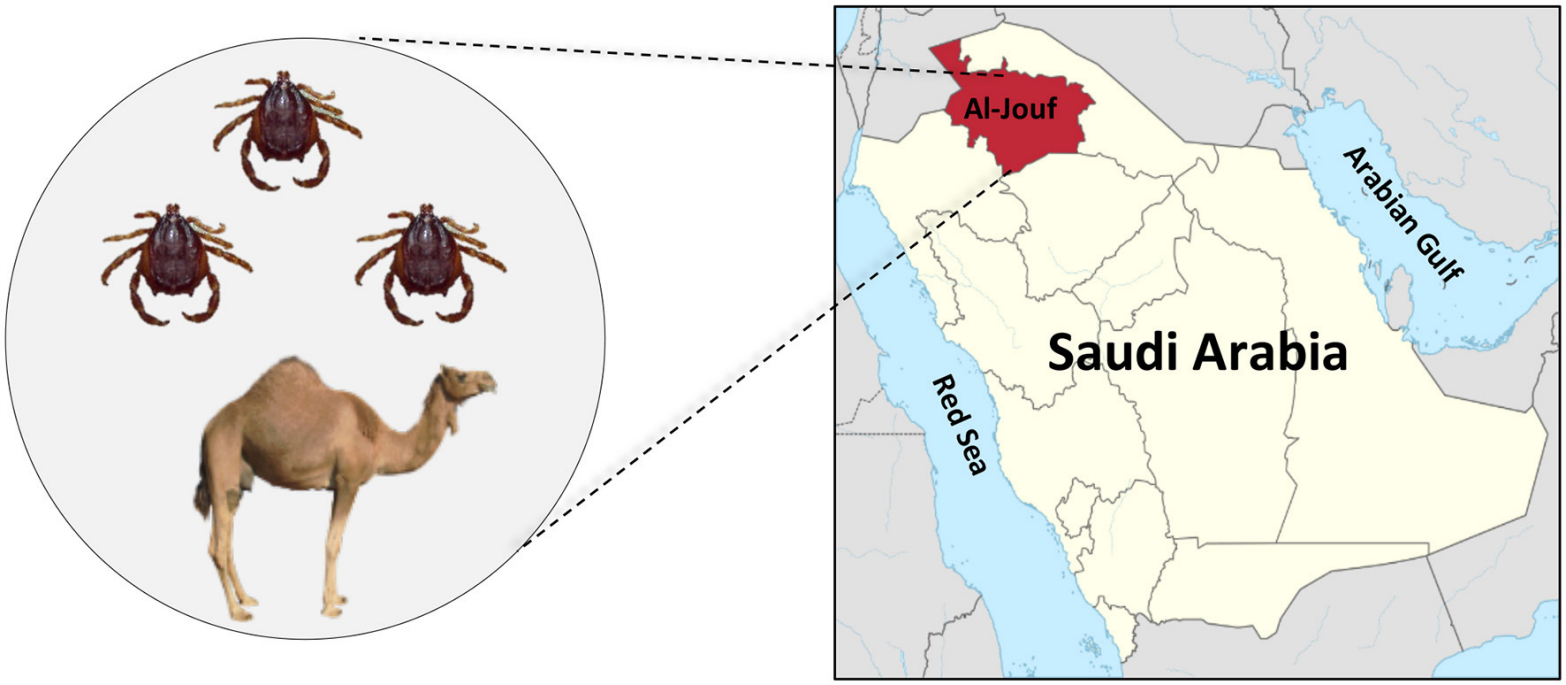
The geographic location of Al-Jouf province within Saudi Arabia where ticks (*H. dromedarii*) were collected from camels.

The current study used hard ticks collected from dromedary camels throughout Al-Jouf province (29.8874° N and 39.3206° E) in the northern part of Saudi Arabia (Figure 1). Al-Jouf region is one of the most fertile regions in Saudi Arabia as a soil and water treasure. In addition, it has a suitable atmosphere which lies between the climate of the Arabian Peninsula and the climate of the Mediterranean Sea. These conditions have made the region suitable for cultivating various crops. Therefore, the agricultural sector occupies most of the economic activities in the Al-Jouf region. With the availability of agricultural resources, the region has developed the livestock sector. Approximately 7,398 camels were recorded in the Al-Jouf region in 2005 (20). These factors made the area suitable for our study due to the availability of a large number of animals and the frequent human-animal interaction.

### 2.2 The collection and identification of the ticks

In total, 49 ticks were collected in August 2022 from 13 female dromedary camels (*Camelus dromedaries*) from Al-Jouf camel market (Supplementary Table 1). The collected ticks were stored in a jar containing 70% ethanol and transported to the Department of Pharmacology and Toxicology, College of Pharmacy, King Saud University, for further analysis. Collected specimens were subjected to taxonomic identification based on the external morphology to the species level, with developing stages and gender recorded using standard morphological keys by means of the stereomicroscope (Leica EZ4HD) (21).

### 2.3 Bacterial isolation from ticks

Ticks were washed with 70% ethanol and rinsed 3 times with phosphate-buffered saline (PBS) (PanReac, AppliChem). Tick's exoskeletons were removed using sterile forceps and blades, and the internal organs were cut into small pieces and then transferred into tubes. Each tick was homogenized individually with PBS using an electric homogenizer. Each homogenate was inoculated into 3 ml of nutrient broth media (VWR Chemicals, USA) in a 15 ml tube and incubated for 24 h at 37°C with shaking (250 rpm) (10, 22). The growing cultures were plated into different media, including blood agar base (VWR Chemicals, USA) and MacConkey agar (OXOID, UK) to allow the growth of a large spectrum of bacteria (23). After 24 h of incubation at 37°C, different colonies (1–2 colonies from each plate) were selected based on morphology (color, structure, shape, size). The bacterial isolates were stored with glycerol at −80°C for further analysis.

### 2.4 Identification of the bacterial isolates

Bacterial isolates were subjected to Gram staining to differentiate the Gram-positive and Gram-negative bacteria (23). After that, bacteria were identified by the Vitek 2 compact system (bioMérieux Inc. USA) (24). The identification was conducted using GP ID REF21342 card (for Gram-positive) and GN ID REF21341 card (for Gram-negative). All procedures were followed according to the manufacturer's instructions.

### 2.5 Determination of antimicrobail agents susceptibility of the bacterial isolates

Antimicrobial susceptibility test was conducted for 45 identified isolates using Vitek 2 compact system (bioMérieux Inc. USA) (23). Antimicrobial susceptibility tests were run on cards that contain dilutions of antimicrobials to detect the breakpoint minimum inhibitory concentration (MIC) against the bacteria. AST-P580 card (for *Staphylococcus* spp., *Enterococcus* spp., and *Streptococcus agalactiae*), and AST-N291 card (for Gram-negative bacilli) cards (bioMérieux Inc. USA) were used to determine antimicrobial agent's susceptibility. The used antimicrobial agents' classes include: penicillins (ampicillin, amoxicillin/clavulanic acid, piperacillin/tazobactam, benzylpenicillin, oxacillin); aminoglycosides (gentamicin, tobramycin, amikacin); cephalosporin (cephalothin, cefoxitin, ceftazidime, ceftriaxone, cefepime); carbapenem (imipenem, meropenem); fluoroquinolone (ciprofloxacin, levofloxacin, moxifloxacin), tetracyclines (tetracycline, tigecycline); glycopeptide (teicoplanin, vancomycin); macrolides (erythromycin); lincomycin (clindamycin); oxazolidinone (linezolid); rifamycin (rifampicin); nitrofuran (nitrofurantoin); and Sulfonamides (trimethoprim/sulfamethoxazole). According to their known or expected primary activity, these agents were tested against either Gram-positive or Gram-negative species. The following quality control strains were included in all tests: *E. coli* ATCC 25922 and 35218, *Staphylococcus aureus* ATCC 29213, *Pseudomonas aeruginosa* ATCC 27853, *Enterococcus faecalis* ATCC 29212, *Haemophilus influenzae* ATCC 49247 and 49766, and *Streptococcus pneumoniae* ATCC 49619. The MIC cutoff values distinguishing sensitive, moderate, and resistant bacteria to antimicrobial agents were programmed into the system per the National Committee for Clinical Laboratory Standards (NCCLS), USA guidelines. The results were interpreted using Vitek 2 compact software version 07.01.

## 3 Results

### 3.1 Identification of the isolated bacteria

The collected 49 ticks from 13 female dromedary camels were classified as *Hyalomma dromedarii* and they were either nymphs (79.6%, *n* = 39) or males (20.4%, *n* = 10). From the collected ticks, a total of 55 bacterial species were isolated and subjected to Gram staining, followed by identification by Vitek 2 compact system. The results showed that the bacterial isolates belong to 20 different bacterial species. About 71% (*n* = 39) of the total isolates were identified as Gram-positive bacteria comprised of 11 different species: *Staphylococcus lentus* (*n* = 11), *Staphylococcus pseudintermedius* (*n* = 9), *Aerococcus viridans* (*n* = 4), *Staphylococcus aureus* (*n* = 3), *Staphylococcus vitulinus* (*n* = 3), *Staphylococcus sciuri* (*n* = 2), *Staphylococcus haemolyticus* (*n* = 2), *Enterococcus casseliflavus* (*n* = 2), *Staphylococcus hominis* (*n* = 1), *Staphylococcus epidermidis* (*n* = 1), and *Streptococcus equi ssp zooepidemicus* (*n* = 1). The Gram-negative bacteria were represented by 29% (*n* = 16) of the total isolates comprised of 9 different species: *Sphingomonas paucimobilis* (*n* = 8), *Klebsiella pneumoniae ssp ozaenae* (*n* = 1)*, Klebsiella pneumoniae ssp pneumoniae* (*n* = 1)*, Pseudomonas aeruginosa* (*n* = 1)*, Pseudomonas putida* (*n* = 1)*, Pseudomonas fluorescens* (*n* = 1), *Stenotrophomonas maltophilia* (*n* = 1), *Rhizobium radiobacter* (*n* = 1) and *Cronobacter sakazakii group* (*n* = 1). The most prevalent isolated bacteria was *S. lentus* (20%, *n* = 11), followed by *S. pseudintermedius* (16.4%, *n* = 9) and *S. paucimobilis* (14.5%, n = 8). Interestingly, each collected tick was found positive for one bacterial specie, however, 4 out of the 49 ticks were found to have 2 or 3 bacterial species (Supplementary Table 1).

### 3.2 Determination of the antimicrobial agents susceptibility of the isolated bacteria

Fourty-five bacterial isolates were tested for antimicrobial susceptibility using Vitek 2 compact system (among them 31 isolates are Gram-positive and 14 isolates are Gram-negative). Our results showed that Gram-positive and Gram-negative bacteria exhibited resistance to several antimicrobial agents (Table 1). The antimicrobial susceptibility profile of Gram-positive bacteria showed that 100% (*n* = 31) were resistant to benzylpenicillin; 90.3% (*n* = 28) were resistant to oxacillin; 58.1% (*n* = 18) were resistant to clindamycin; 48.4% (*n* = 15) were resistant to vancomycin. In addition, 32.3% (*n* = 10) were resistant to trimethoprim/sulfamethoxazole and rifampicin; 25.8% (*n* = 8) were resistant to erythromycin; 16.1% (*n* = 5) were resistant to teicoplanin; 6.5% (*n* = 2) were resistant to tetracycline (Table 1). All Gram-positive bacteria were 100% susceptible to linezolid, gentamicin, tobramycin, levofloxacin, moxifloxacin, tigecycline, and nitrofurantoin (Table 1).

**Table 1:** Antimicrobials susceptibility of Gram-positive and Gram-negative bacteria isolated from *H. dromedarii*.

**Gram-positive bacteria**																
**Pattern**	**Benzylpenicillin**	**Oxacillin**	**Gentamicin**	**Tobramycin**	**Levofloxacin**	**Moxifloxacin**	**Erythromycin**	**Clindamycin**	**Linezolid**	**Teicoplanin**	**Vancomycin**	**Tetracycline**	**Tigecycline**	**Nitrofurantoin**	**Rifampicin**	**Trimethoprim/sulfamethoxazole**
**S**	0	9.7% (3)	100% (31)	100% (31)	100% (31)	100% (31)	35.5% (11)	38.7% (12)	97% (30)	83.9% (26)	51.6% (16)	83.9% (26)	100% (31)	93.5% (29)	67.7% (21)	67.7% (21)
**I**	0	0	0	0	0	0	38.7% (12)	3.2% (1)	3% (1)	0	0	9.6% (3)	0	6.5% (2)	0	0
**R**	100% (31)	90.3% (28)	0	0	0	0	25.8% (8)	58.1% (18)	0	16.1% (5)	48.4% (15)	6.5% (2)	0	0	32.3% (10)	32.3% (10)
**Gram-negative bacteria**																
**Pattern**	**Ampicillin**	**Amoxicillin/ clavulanic acid**	**Piperacillin/tazobactam**	**Cefalotin**	**Cefoxitin**	**Ceftazidime**	**Ceftriaxone**	**Cefepime**	**Imipenem**	**Meropenem**	**Amikacin**	**Gentamicin**	**Ciprofloxacin**	**Tigecycline**	**Nitrofurantoin**	**Trimethoprim/sulfamethoxazole**
**S**	28.57% (4)	71.43% (10)	92.86% (13)	78.57% (11)	50% (7)	42.86% (6)	42.86% (6)	85.71% (12)	100% (14)	100% (14)	100% (14)	100% (14)	100% (14)	92.86% (13)	78.57% (11)	71.43% (10)
**I**	14.29% (2)	7.14% (1)	0	0	0	7.14% (1)	28.57% (4)	0	0	0	0	0	0	0	7.14% (1)	0
**R**	57.14% (8)	21.43% (3)	7.14% (1)	21.43% (3)	50% (7)	50% (7)	28.57% (4)	14.29% (2)	0	0	0	0	0	7.14% (1)	14.29% (2)	28.57% (4)

S, susceptible; I, intermediate resistant; R, resistant. The numbers in parentheses indicate the number of bacteria.

For the Gram-negative bacterial antimicrobial agents susceptibility testing, 57.14% (*n* = 8) of the identified Gram-negative bacteria were resistant to ampicillin, whereas 50% (*n* = 7) were resistant to cefoxitin and ceftazidime. About 28.57% (*n* = 4) of the Gram-negative bacteria were resistant to ceftriaxone, trimethoprim/sulfamethoxazole. In addition, 21.43% (*n* = 3) were resistant to amoxicillin/clavulanic acid and cephalothin; 14.29% (*n* = 2) were resistant to cefepime and nitrofurantoin; 7.14% (*n* = 1) were resistant to piperacillin/tazobactam and tigecycline (Table 1). However, all Gram-negative bacteria were susceptible to other antimicrobials including imipenem, meropenem, amikacin, gentamicin, and ciprofloxacin (Table 1).

**Figure 2:**
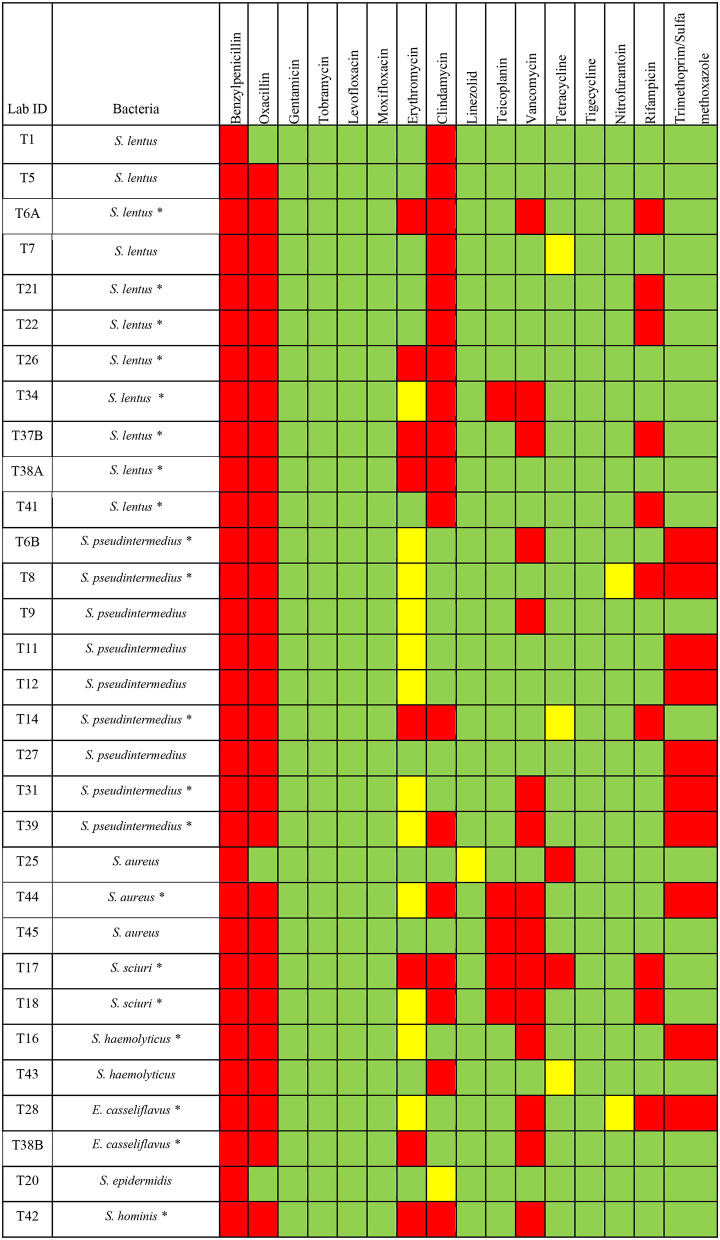
Antimicrobials susceptibility of different Gram-positive bacteria isolated from *H. dromedarii*. Green, susceptible; Yellow, intermediate resistant; Red, resistant. The star symbol next to the bacteria name denotes the MDR bacteria.

All Gram-positive bacteria tested for antimicrobial agents susceptibility showed resistance to one or more classes of antimicrobials (Figure 2). Among *S. lentus* isolates, all isolates (*n* = 11/11) showed resistance to benzylpenicillin and clindamycin, 10 isolates (*n* = 10/11) showed resistance to oxacillin, 5 isolates (*n* = 5/11) showed resistance to rifampicin, 4 isolates (*n* = 4/11) showed resistance to erythromycin, 3 isolates (*n* = 3/11) showed resistance to vancomycin, only one isolate of *S. lentus* showed resistance to teicoplanin (Figure 2). All *S. pseudintermedius* isolates (*n* = 9/9) showed resistance to benzylpenicillin and oxacillin, 7 isolates (*n* = 7/9) showed resistance to trimethoprim/sulfamethoxazole, 4 isolates (*n* = 4/9) showed resistance to vancomycin, 2 isolates (*n* = 2/9) showed resistance to rifampicin and clindamycin, only one isolate (*n* = 1/9) showed resistance to erythromycin (Figure 2). For the *S. aurues*, all isolates (*n* = 3/3) showed resistance to benzylpenicillin, two isolates (*n* = 2/3) showed resistance to oxacillin, teicoplanin and vancomycin, and only one isolate of *S. aurues* (*n* = 1/3) showed resistance to clindamycin, tetracycline and trimethoprim/sulfamethoxazole (Figure 2). For *S. sciuri, two* isolates (*n* = 2/2) showed resistance to benzylpenicillin, oxacillin, clindamycin, teicoplanin, vancomycin and rifampicin, one isolate (*n* = 1/2) showed resistance to erythromycin and tetracycline. *E. casseliflavus* isolates (*n* = 2/2) showed resistance to benzylpenicillin, oxacillin, and vancomycin. One isolate of *E. casseliflavus* (*n* = 1/2) showed resistance to erythromycin, rifampicin, and trimethoprim/sulfamethoxazole (Figure 2). For *S. haemolyticus*, two isolates (*n* = 2/2) showed resistance to benzylpenicillin and oxacillin, one isolate (*n* = 1/2) showed resistance to clindamycin. *S. hominis* isolate showed resistance to benzylpenicillin, oxacillin, erythromycin, clindamycin, and vancomycin. *S. epidermidis* showed resistance to benzylpenicillin.

**Figure 3:**
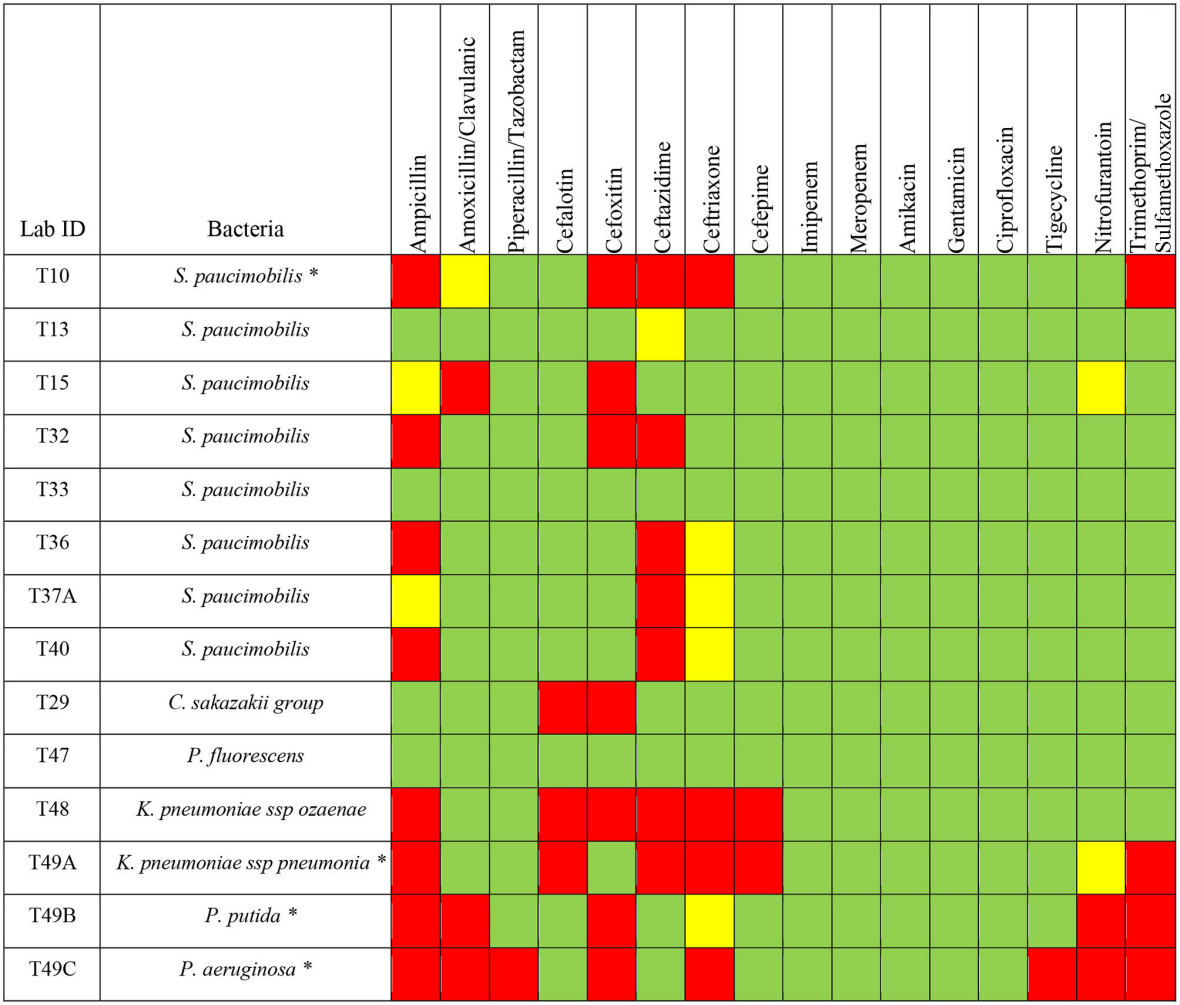
Antimicrobials susceptibility of different Gram-negative bacteria isolated from *H. dromedarii*. Green, susceptible; Yellow, intermediate resistant; Red, resistant. The star symbol next to the bacteria name denotes the MDR bacteria.

Among the Gram-negative bacterial species tested for susceptibility, 11 out of 14 isolates showed resistance to one or more classes of antimicrobials. For *S. paucimobilis*, 5 isolates (*n* = 5/8) showed resistance to ceftazidime, 4 isolates (*n* = 4/8) showed resistance to ampicillin, 3 isolates (*n* = 3/8) showed resistance to cefoxitin. Only one isolate of *S. paucimobilis* (*n* = 1/8) showed resistance to amoxicillin/clavulanic acid, ceftriaxone, and trimethoprim/sulfamethoxazole (Figure 3). *C. sakazakii group* showed resistance to cefoxitin and cefalotin. *K. pneumoniae ssp ozaenae* showed resistance to ampicillin, cephalothin, cefoxitin, ceftazidime, ceftriaxone, cefepime. *K. pneumoniae ssp pneumonia* showed resistance to ampicillin, cephalothin, ceftazidime, ceftriaxone, cefepime, and trimethoprim/sulfamethoxazole. *P. putida* showed resistance to ampicillin, amoxicillin/clavulanic acid, cefoxitin, nitrofurantoin, and trimethoprim/sulfamethoxazole. *P. aeruginosa* showed resistance to ampicillin, amoxicillin/clavulanic acid, piperacillin/tazobactam, cefoxitin, ceftriaxone, tigecycline, nitrofurantoin and trimethoprim/sulfamethoxazole (Figure 3).

For MDR bacteria, we looked for any bacterial isolate that is resistant to three or more of the antimicrobial agent classes. Among the Gram-positive bacteria, we found that 64.5% (*n* = 20) are considered as MDR bacteria with some isolates resistant to even more than 4 classes of antimicrobial agents (Figure 2). The percentage of MDR bacteria among the Gram-positive bacterial species was as the following: *S. lentus* (72.7%, *n* = 8/11), *S. pseudintermedius* (55.5%, *n* = 5/9), *S. sciuri* (100%, *n* = 2/2), *E. casseliflavus* (100%, *n* = 2/2), *S. hominis* (100%, *n* = 1/1), *S. aurues* (33.3%, *n* = 1/3), *S. haemolyticus* (50%, *n* = 1/2) (Figure 2).

Among the Gram-negative bacteria, we found that 28.5% (*n* = 4) are MDR bacteria with some isolates resistant to even more than 4 classes of antimicrobial agents (Figure 3). One isolate of *S. paucimobilis* (*n* = 1/8), *K. pneumoniae ssp pneumoniae, P. aeruginosa*, and *P. putida*, showed resistance to 3 or more different classes of antimicrobials (Figure 3).

## 4 Discussion

The increasing prevalence of AMR bacteria is a global concern affecting both animal and human health. The role of ticks in disseminating AMR bacteria is not completely understood. To address this issue, different bacteria were isolated from *H. dromedarii* ticks from Al-Jouf province and their antimicrobial susceptibilities to different clinically utilized antimicrobials were determined. We concluded that isolated bacteria from ticks showed significant resistance to different antimicrobial agents, including benzylpenicillin; oxacillin, clindamycin, vancomycin, trimethoprim/sulfamethoxazole, rifampicin, erythromycin, teicoplanin, tetracycline, ampicillin, cefoxitin, ceftazidime, ceftriaxone, trimethoprim/sulfamethoxazole, amoxicillin/clavulanic acid, cephalothin, cefepime and nitrofurantoin, piperacillin/tazobactam, and tigecycline. Collectively, these data indicate the possibility of the existence of different AMR bacteria within the ticks that needs further research impetus. Furthermore, almost all isolated bacterial species can infect and spread between animals and humans, causing economic and health-related problems (25–31). To our knowledge, this is the first report in the region about ticks and their association with AMR bacteria.

During our study, several genera of Gram-positive and Gram-negative bacteria were identified which included species of the genera of *Staphylococcus, Enterococcus, Streptococcus, Klebsiella Pseudomonas*, and *Stenotrophomonas* which is similar to the findings from other studies (32–34). Some of these bacteria of the genera of *Enterococcus, Streptococcus, Pseudomonas*, and *Klebsiella* are potential pathogens to both humans and animals. Ticks may play a role as reservoir hosts for pathogenic bacteria leading to public and veterinary health risks. Other tick species may be screened for AMR bacteria to minimize any zoonotic consequences.

About 42 bacterial samples isolated from the ticks showed resistance to one or more antimicrobial agents from 9 different classes including pipracillins (ampicillin, amoxicillin/clavulanic acid, piperacillin/tazobactam, benzylpenicillin, oxacillin); cephalosporin (cephalothin, cefoxitin, ceftazidime, ceftriaxone, cefepime); tetracyclines (tetracycline, tigecycline); glycopeptide (teicoplanin, vancomycin); macrolides (erythromycin); lincomycin (clindamycin); rifamycin (rifampicin); nitrofuran (nitrofurantoin); and Sulfonamides (trimethoprim/sulfamethoxazole). Interestingly, among the 42 AMR bacteria, 24 bacterial isolates were resistant to 3 or more antimicrobials, considering MDR bacteria. This needs further research to investigate antimicrobial-resistant genes (ARGs) against the tested antimicrobial agents. The observed resistant phenotypes are concerning and supporting the active role of ticks as carriers of AMR bacteria. Scientific research has already established a direct correlation between antimicrobial use and the degree of resistance (35, 36). However, further studies must examine the correlation between microbiota, ARGs, and antimicrobial use in ticks. Monitoring efforts must be further emphasized where antimicrobials are widely utilized and tick-borne diseases are endemic. Even our results showed that ticks might act as a vector that transmits different AMR bacteria. Further studies are essential to answer questions related to the role of ticks in the spread and transmission of AMR bacteria among different hosts, including humans, animals, and the environment.

Tick microbiota could be influenced by the environment and blood meals (37, 38). The environment contains many species of bacterial genera, for example: *Staphylococcus* and *Pseudomonas*. We found these bacterial genera in ticks, which might suggest that these bacteria were acquired by the ticks from the surrounding environment. Previous studies have also observed the presence of these bacterial genera in ticks. However, there are still debates whether these bacteria are just environmental contaminants, or they are belonging to the tick microbiota (39–41).

The tick-host interaction facilitates the transmission of AMR bacteria between the ticks and their hosts (42). During our study, different AMR bacteria were identified and some of them could be potential pathogens. On the other hand, we identified different species of the *Staphylococcus* including *S. aureus*, which could be part of the tick's microbiota. However, there is a possibility that *Staphylococcus* spp. has been acquired from other hosts or environments since they are resistant to different classes of antimicrobial agents. The presence of different bacteria within the ticks combined with different resistance patterns could indicate the active transmission of these AMR bacteria between the ticks and their hosts. Further studies should be encouraged to determine the role of ticks in the transmission of AMR bacteria to animals as well as humans.

## 5 Conclusion

Our study highlighted the risk of camels' ticks as a reservoir for AMR bacteria. There is a significant risk of transmitting AMR bacteria among camels, humans, and other animals that are meditated through ticks leading to a public health concern. This study will lay a foundation for future research on AMR pathogens transmitted by ticks and to increase the awareness of tick-transmitted pathogens that threaten the public and animal health.

## Data availability statement

The original contributions presented in the study are included in the article/Supplementary material, further inquiries can be directed to the corresponding author.

## Author contributions

Conceptualization: MMA, AAlo, and AAli. Methodology: ATA, MMA NA, AOA, and ED. Validation: MMA, AAlo, and WA. Analysis: ATA, MMA, ED, MA, TT, and AAli. Resources: MMA. Writing—original draft preparation: ATA, MMA, and AAli. Writing—review and editing: ATA, MMA, AAlo, AAli, and AOA. All authors have read and agreed to the published version of the manuscript.
