# Supplementary material for: Isolation, identification and antimicrobial susceptibility of the bacteria isolated from Hyalomma dromedarii infesting camels in Al-Jouf province, Saudi Arabia
Source: Front Vet Sci. 2023 Dec 14;10:1227908. doi: 10.3389/fvets.2023.1227908 (PMC10752931; doi:10.3389/fvets.2023.1227908)
Supplement: Supplementary file 1 [file Table_1.docx]

**Supplementary Table 1: Information about the number of** ***Hyalomma dromedarii* ticks that have been collected from different camels and the bacterial isolates from each tick.**

| **Camel number** | **Tick number** | **Gender of Ticks** | **Isolated Bacteria** | Lab ID |
| --- | --- | --- | --- | --- |
| **01** | **1** | Male | *S. lentus* | T1 |
|  | **2** | Nymph | *A. viridans* | T2 |
|  | **3** | Nymph | *A. viridans* | T3 |
|  | **4** | Nymph | *S. vitulinus* | T4 |
|  | **5** | Male | *S. lentus* | T5 |
| **02** | **6** | Nymph | *S. lentus* | T6A |
|  |  |  | *S. pseudintermedius* | T6B |
|  | **7** | Male | *S.lentus* | T7 |
|  | **8** | Nymph | *S. pseudintermedius* | T8 |
|  | **9** | Nymph | *S.pseudintermedius* | T9 |
| **03** | **10** | Nymph | *S. paucimobilis* | T10 |
|  | **11** | Male | *S. pseudintermedius* | T11 |
|  | **12** | Male | *S. pseudintermedius* | T12 |
|  | **13** | Nymph | *S. paucimobilis* | T13 |
|  | **14** | Nymph | *S. pseudintermedius* | T14 |
|  | **15** | Nymph | *S. paucimobilis* | T15 |
| **04** | **16** | Nymph | *S haemolyticus* | T16 |
|  | **17** | Nymph | *S. sciuri* | T17 |
|  | **18** | Nymph | *S. sciuri* | T18 |
| **05** | **19** | Nymph | *S. vitulinus* | T19 |
|  | **20** | Male | *S. epidermidis* | T20 |
|  | **21** | Nymph | *S. lentus* | T21 |
|  | **22** | Nymph | *S. lentus* | T22 |
| **06** | **23** | Nymph | *R. radiobacter* | T23 |
|  | **24** | Male | *A. viridans* | 24 |
|  | **25** | Nymph | *S. aureus* | T25 |
|  | **26** | Nymph | *S. lentus* | T26 |
|  | **27** | Male | *S. pseudintermedius* | T27 |
| **07** | **28** | Nymph | *E. casseliflavus* | T28 |
|  | **29** | Nymph | *C. sakazakii group* | T29 |
|  | **30** | Nymph | *S. vitulinus* | T30 |
| **08** | **31** | Nymph | *S. pseudintermedius* | T31 |
|  | **32** | Nymph | *S. paucimobilis* | T32 |
|  | **33** | Male | *S. paucimobilis* | T33 |
|  | **34** | Nymph | *S. lentus* | T34 |
| **09** | **35** | Nymph | *S. equi ssp zooepidemicus* | T35 |
|  | **36** | Nymph | *S. paucimobillis* | T36 |
|  | **37** | Nymph | *S. paucimobilis* | T37A |
|  |  |  | *S. lentus* | T37B |
|  |  |  | *A. viridans* | T37C |
| **010** | **38** | Nymph | *S. lentus* | T38A |
|  |  |  | *E. casseliflavus* | T38B |
|  | **39** | Nymph | *S. pseudintermedius* | T39 |
| **011** | **40** | Nymph | *S. paucimobillis* | T40 |
|  | **41** | Nymph | *S. lentus* | T41 |
|  | **42** | Nymph | *S. hominis ssp hominis* | T42 |
| **012** | **43** | Male | *S. haemolyticus* | T43 |
|  | **44** | Nymph | *S. aureus* | T44 |
|  | **45** | Nymph | *S. aureus* | T45 |
|  | **46** | Nymph | *S. maltophilia* | T46 |
| **013** | **47** | Nymph | *P. fluorescens* | T47 |
|  | **48** | Nymph | *K. pneumoniae ssp ozaenae* | T48 |
|  | **49** | Nymph | *K. pneumoniae* | T49A |
|  |  |  | *P. putida* | T49B |
|  |  |  | *P. aeruginosa* | T49C |
